# Anterior Prefrontal Contributions to Implicit Attention Control

**DOI:** 10.3390/brainsci2020254

**Published:** 2012-06-15

**Authors:** Stefan Pollmann

**Affiliations:** Experimental Psychology Lab, Institute of Psychology II, Otto-von-Guericke-University, Postbox 4120, D-39016 Magdeburg, Germany; E-Mail: stefan.pollmann@ovgu.de; Tel.: +49-391-6718474; Fax: +49-391-6711401

**Keywords:** prefrontal, frontopolar, implicit, learning, unconscious

## Abstract

Prefrontal cortex function has traditionally been associated with explicit executive function. Recently, however, evidence has been presented that lateral prefrontal cortex is also involved in high-level cognitive processes such as task set selection or inhibition in the absence of awareness. Here, we discuss evidence that not only lateral prefrontal cortex, but also rostral prefrontal cortex is involved in such kinds of implicit control processes. Specifically, rostral prefrontal cortex activation changes have been observed when implicitly learned spatial contingencies in a search display become invalid, requiring a change of attentional settings for optimal guidance of visual search.

## 1. Introduction

In the current paper, I selectively review evidence for an involvement of anterior prefrontal cortex in implicit attention control. The paper is not an exhaustive review of anterior prefrontal function, which has been covered in several excellent reviews in recent years [1,2]. There is ample evidence that anterior prefrontal areas support high-level executive functions. However, executive processes are typically thought of as volitional processes, carried out by the individual in awareness.

However, prefrontal, and specifically rostral prefrontal cortex activation has repeatedly been observed in situations which require implicit changes of attentional settings [3,4]; Recently, several studies have additionally provided evidence for prefrontal involvement in high-level executive processing in the absence of awareness [5]. 

## 2. Prefrontal Involvement in High-Level Cognition in the Absence of Awareness

For some time, there has been debate as to whether prefrontal cortex is necessary for consciousness [6]. Proponents of this idea argue for long-range connections between posterior sensory areas and prefrontal cortex [7,8,9,10,11], whereas others note that prefrontal lesions do not typically lead to impairment of conscious perception [12]. The hypothesis that prefrontal cortex is necessary for conscious perception and action fits nicely with the longstanding view of prefrontal cortex as a necessary substrate of high-level executive function. More recently, however, the reverse question has gained importance, namely in how far prefrontal cortex supports implicit processing in domains which traditionally have been considered to be exclusively under explicit volitional control. Lau and Passingham [13] showed a mid-dorsolateral prefrontal response to incongruous primes that were not consciously perceived (interestingly, this congruency effect was not observed for visible primes). They used a metacontrast mask (for the properties of this kind of masking see Vorberg *et al*. [14]) to indicate the task instruction, a phonological *versus* semantic judgment of a visually presented word. Similarly, van Gaal *et al*. [15] found that inhibitory primes, made invisible by metacontrast masking, elicited electrophysiological potential changes over occipital and frontocentral leads. The occipital potential, occurring earlier (~150 ms post stimulus onset), represented stimulus encoding and was observed independently of the prime’s task relevance. The frontal potential, occurring somewhat later (roughly 300–400 ms post stimulus onset), was specifically associated with the inhibitory control in the go/no-go experiment. Source reconstruction indicated the bilateral (somewhat right-dominant) anterior prefrontal cortex as potential “generator” site. A later fMRI-experiment from the same group [16] reported activation associated with unconscious no-go commands in region-of-interest analyses of the inferior frontal gyrus in the vicinity of the anterior insula as well as the pre-supplementary motor area. In the absence of a whole brain analysis of this contrast, it remained unclear how well the source reconstruction of the electrophysiological data and the fMRI activation corresponded. 

Thus, both selection of a task set [13] and inhibition of a task set [15,16] were induced by primes that were not consciously perceived. Furthermore, both unconscious task set selection and task set inhibition were associated with prefrontal activity. Currently, one can only speculate whether the involvement of more anterior prefrontal sites suggested by the source reconstruction of van Gaal *et al*. [15] was specifically tied to inhibition and the mid-dorsolateral activation to task set selection, as both studies used somewhat different experimental paradigms. 

Further evidence for an interaction of implicit low-level visual and high-level attentional processing comes from studies of perceptual learning. Tsushima *et al*. [17] tested perceptual learning of motion direction stimuli during an attention-demanding rapid serial visual presentation (RSVP) task. Interestingly, they found that the task-irrelevant motion stimuli led to perceptual learning when the coherence of moving dots was low, leading to near threshold performance, while no learning occurred for high coherence suprathreshold moving dots. They further observed that fMRI-activation in lateral prefrontal cortex was inversely correlated with activation in the motion processing area hMT+. While hMT+ activation increased for lower thresholds, the reverse was observed for lateral prefrontal cortex. The authors suggested that prefrontal cortex, involved in inhibition of task-irrelevant processing, may have a higher threshold for processing motion-direction than a specialized area such as human MT+. Consequently, prefrontally guided inhibition of the irrelevant motion stimuli occurred only for suprathreshold stimuli. This, however, does not mean that subliminal perceptual learning proceeds completely free of attentional resources. Combining the attentional blink paradigm with the motion direction perceptual learning task, Seitz *et al*. [18] could show that subliminal perceptual learning was reduced during the blink. What exactly was contributed by attentional processing remained unspecified, however, it appeared not to be attentive processing of to be learned features (motion direction, 5% coherence) *per se*. A possible attentional function is “stimulus tagging”, *i.e*., the association of to be learned features with a featural or temporal cue [19,20]. According to the stimulus tagging model, the cue can be conceptual or semantic, involving prefrontal processing. This processing is thought to be a top-down modulation of posterior visual areas. 

In another line of research, evidence was provided that conflict resolution in the Stroop [21] or Flanker tasks [22] may occur to a large degree on the level of individual items instead of a global control setting. For instance, in the Stroop task, conflict arises when subjects are asked to name the print color of the word and the word denotes a different color (e.g., the word “red” printed in green). In this case, responses are slower than for congruent color words. The size of this effect, however, depends on the frequency of congruent words. If the number of incongruent trials is high, the difference between incongruent and congruent response times is smaller than if it is low. Usually, this is interpreted as top-down controlled, strategic processing [23,24]. However, it could be shown that this proportion effect is independent of explicit ratings of the proportion of incongruent trials, making it difficult to assume that the effect is caused by deliberate strategic control [25]. This proportion effect can be taken to the level of individual stimuli (e.g., blue items are associated with frequent, red items with rare conflicts [26]). That is, conflict adaptation occurs not at the level of rules that are consistently held over the duration of an experiment or strategic adaptations to the overall congruency proportion over a block of trials but rather based on congruency proportions associated with particular features. The latter are likely to be learned implicitly and may not reach awareness [27]. Thus, what seemed like driven by global strategic control was rather driven by local stimulus driven adaptation. Using this proportion congruent variation in a Stroop task, prefrontal activations observed in conflict trials appeared to support local rather than global cognitive control [28]. The activation changes tied to local control were observed mostly in posterior parts of prefrontal cortex. One “local control”-activation bordered anterior prefrontal cortex (MNI coordinates −39, 51, 24), whereas the only truly anterior prefrontal activation change (at −27, 60, 27) appeared to support global control. Similarly, in a study investigating adjustment to different conflict ratios tied to specific locations, responses to these differences (e.g., indicative of “local control” were observed in lateral and medial prefrontal cortex, whereas the only BA 10 (*i.e*., anterior prefrontal) activation was observed in the “global control” condition without local differences in conflict ratios [29]. This activation, however, was observed in the right hemisphere (14, 64, 12), in contrast to the anterior prefrontal activation in the study by Blais and Bunge [28]. It remains an interesting question whether anterior-posterior distinctions between global and local control can be replicated across different paradigms. 

All of the above studies (with the possible exception of van Gaal *et al*. [15]) implied structures of the lateral prefrontal cortex in implicit cognitive control that are posterior to frontopolar cortex. This rostral part of prefrontal cortex has been associated with high-level executive processes involving timing of and resource allocation between several stimulus aspects or tasks, like integration of multiple relations [30]; subgoal processing [31], cognitive branching [2,32], switching between internally and externally generated information [33,34] or prospective memory [35]. In the remainder, we discuss evidence suggesting that frontopolar cortex is also involved in high-level executive processes that occur in the absence of awareness. 

## 3. Frontopolar Involvement in the Implicit Control of Attention

### 3.1. Cross-Dimensional Visual Singleton Search

Previous studies from my lab have shown that specifically the left lateral frontopolar cortex is consistently activated during attention changes between spatial locations or visual dimensions (reviewed in [3]). These attention change-related activations were mostly observed when on the one hand the reallocation of attention was adaptive but on the other hand there were no clear rules when or where to change attention. As an example, frontopolar activation was observed following changes of the target-defining dimension in visual search for singleton targets [36,37]. In these experiments, targets were defined by a unique color or motion direction in the search display. Behavioral data showed costs following target dimension changes, suggesting a re-weighting of attentional resources from the old to the new dimension [38]. This interpretation was supported by increased activation in visual areas processing the current target dimension (V4 for color, hMT+ for motion; [39]). There was also a selective dimension change cost in patients with left lateral frontopolar lesions, underlining the functional contribution of this area [40]. Thus, in singleton search, shifts of attention occur in the absence of explicit instructions to shift attention between visual dimensions such as color and motion. Moreover, since it is a target detection task, the same response is carried out on singletons, independent of their defining dimension (unique color, motion, *etc*.). This led us to believe that the shifts of attention from the old to the new dimension in this task may be carried out in the absence of awareness. We could, however, not prove this claim in the singleton feature search task. This is why we turned to the contextual cueing paradigm. 

### 3.2. Contextual Cueing

Adjustments of attention may also occur to changes in the environment that the individual is unaware of. One such situation is contextual cueing in repeated visual search displays [41]. In this paradigm, a subset of search displays is repeatedly presented throughout the experiment, leading to faster search times in the repeated displays compared to novel displays. Importantly, explicit recognition tests typically yielded no evidence of explicit recognition of the repeated displays, so that contextual cueing is regarded an implicit form of learning (Recently, it has been shown that some displays may also be remembered explicitly, but the size of the contextual cueing effect is typically not related to the degree of explicit performance; [42,43,44]). In order to investigate whether anterior prefrontal cortex is involved in implicit attention changes, we modified the contextual cueing paradigm in that we changed the target position in a repeated distractor configuration after the configuration had been learned. Predictably, the search time advantage for repeated displays was lost after the target location change. Likewise, effects of display repetition on eye movement parameters like number of fixations—reduced for repeated displays—and scan path length—shorter for repeated displays—were lost after target location change [45]. Immediately after the target location change, eye gaze tended to visit the old (now empty) target location. This tendency, however, was only observed in the first block with changed target locations. 

**Figure 1:**
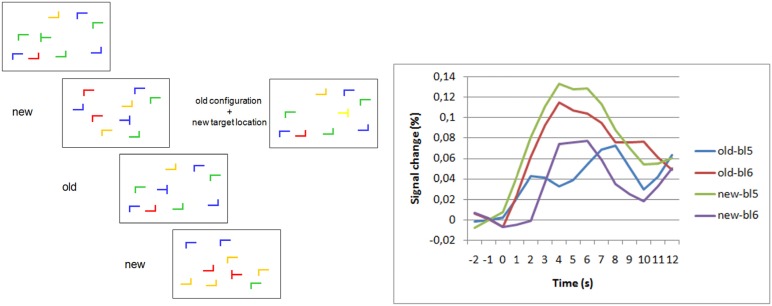
Frontopolar activation in repeated search contexts and target change. (**Left**) Schematic examples of search displays. “New” and “old” labels indicate novel and repeated configurations of “L”-shaped distractors. On the right, a display with repeated distractor configuration but new target location is shown. (**Right**) Blood oxygenation level dependent (BOLD) signal changes for old and new displays in the last block before (bl5) and the first block after (bl6) target location change [46]. Group averaged BOLD-responses are shown after subtraction of null-event responses. The data show a selective increase of the BOLD-response to old displays after the target location change. There was no increase (actually a decrease) in activation to new displays after target location changes. The selective increase of activation for post-change old displays shows the association of the response with the violation of target-location predictiveness of the repeated displays.

Thus, we had evidence that the change of the target location in repeated display configurations had quickly been processed (without becoming aware) and led to the elimination of the search bias towards the old target location. We then ran an fMRI-version of the contextual change paradigm [46]. As expected, we observed a selective increase of activation for old displays after the target location change in left lateral frontopolar cortex (Figure 1). When we split our sample in half by strength of the contextual cueing effect, the frontopolar increase was stronger for the strong than the weak contextual cueing subgroup. The increase of activation was not simply a nonspecific effect due to longer search times, as correlation analyses showed. The explicit recognition test carried out at the end of the scanner session yielded no indication of explicit recognition of repeated displays. 

Among the areas beyond frontopolar cortex that showed a selective post-change increase for repeated displays was the posterior section of the left middle temporal gyrus. The temporoparietal junction area (TPJ) has been discussed to be vital for disengagement of attention from a current attention focus [47]. This might explain post-change TPJ-activation. However, a closer look showed that frontopolar activation reached its maximum in the first post-change block, whereas TPJ activation peaked in the second post-change block. Every block contained 12 different “old” displays (+12 novel displays), which were repeated in each block. Therefore, in the first post-change block, subjects encountered 12 different repeated displays in which for the first time in the experiment, the target was not at the location predicted by the distractor configuration. Thus, the first post-change block offered the opportunity to “detect” (implicitly) the violation of the spatial contingencies between distractor configuration and target location. Only the second post-change block offered the chance to react to these violations, because in this block the 12 displays with old distractor but new target location were presented for the first time after violation detection. Thus, the sequence of frontopolar activation peaking in the first and TPJ activation in the second post-change block may tentatively be interpreted as frontopolar involvement in the implicit “detection” of contingency changes and subsequently stronger TPJ-involvement in the disengagement of attention from the now invalid distractor configuration cues. Certainly this is only a post-hoc hypothesis, but it makes clear predictions for future experiments. 

A relay station to connect rostral prefrontal cortex to posterior areas in implicit control of attention could be inferior frontal gyrus, a structure that is discussed as part of the “reorienting system” of the brain [48]. Inferior frontal activation increase was associated with unconsciously perceived stop signals [16]. Repeated contexts in the contextual cueing paradigm elicited faster fMRI activation in inferior frontal gyrus than novel contexts [49]. Inferior frontal gyrus further shows repetition suppression in priming studies [50,51]. 

In the experiment just described, subjects had a whole training session in the lab before they went to the scanner the next day, continuing search with the same set of repeated displays. These displays were thus well learned when the target location change occurred. In a further experiment, we wanted to investigate whether anterior prefrontal activation following contextual change depends on well-learned contexts or is present already at the beginning of learning [52]. To this end, the repeated displays were presented only six times before the target change occurred. This was not enough to observe a significant contextual cueing effect for repeated displays (which typically needed 12 repetitions with the setup we used), but it should be sufficient to capture the early stage of implicit learning. We again observed contextual change-related activation in frontopolar cortex, this time bilaterally. These data show that the target-distractor contingencies are learned beginning with the first repetitions and anterior prefrontal cortex responds to violations of these contingencies early on. 

### 3.3. Relation to Theoretical Accounts of Anterior Prefrontal Cortex Function

One popular account of frontopolar function attributes it a‚ “branching” function [32]. Branching is similar to task switching with the additional constraint that the current task is not completed before it is interrupted by a subsequent task. Thus, to successfully complete the first task, one must recall its mental state at the time of interruption. An example would be that you have a conversation with a friend, which is interrupted by a telephone call, and you know how (with what topic) to continue the conversation after you have finished the call. This distinguishes branching from task switching, where only two alternative task sets need to be remembered. Thus branching has a stronger contextual element, just as in the contextual cueing studies, in that current stimuli need to be manipulated according to the rules of one task when the rules (and stimuli) of the previous task are still held available. The same holds in general for situations of subgoal processing, which also lead to lateral frontopolar activation (e.g., [31]). A similar situation, which also leads to frontopolar activation, is relational integration [30] Relational integration is characterized by two contexts, e.g., the lines and rows of Raven matrices, which first need to be processed separately and then being integrated to solve the task. All of these tasks have a strong episodic memory component in common. A recent review showed that episodic memory demands consistently activated lateral frontopolar cortex while multitasking activated the more anteromedial parts of frontopolar cortex [1]. This makes it likely that lateral frontopolar cortex plays a role in processing the discrepancy between the contextual memory trace and the actual stimulus input (e.g., change of the target defining dimension in visual singleton search or change of the target location in a repeated search display). Similarly, lateral frontopolar cortex has been reported to respond differentially to the validity of working memory contents used in visual search [53]. Integration of two episodic memory contents has been postulated to be a function specifically of left lateral frontopolar cortex [54]. Moreover, left frontopolar cortex was found to be sensitive to the repeated encoding of an item [55]. Ranganath and colleagues regarded it likely that left frontopolar cortex was involved in the selection and evaluation of specific memory characteristics, a view that fits well with our view of frontopolar contributions to attention control. In the case of contextual cueing, it could be the evaluation whether a repeated context is still predictive of a target location. 

The involvement of frontopolar cortex in situations of contextual interference was very clearly shown in a study by King *et al*. [56], in which different events were shown in virtual reality environments. In one version of the experiment, each event was shown in a separate environment, whereas in another version, the same event occurred in several environments. The most striking difference in activation was observed in frontopolar cortex, which showed significantly stronger activation in the contextual interference condition. This is of course very similar to the repeated context displays with changed target location, with the difference that King and colleagues used an explicit memory task.

In summary, the proposed role of lateral frontopolar cortex in detecting contextual change and potentially suppressing the old context may thus specify one aspect of frontopolar function that falls into the broad concepts of evaluating self-generated information [33], a “gate-keeper” function between stimulus-oriented and stimulus-independent processes [34], or the integration of several mental processes [30,57]. But again, the decisive difference is that these concepts were developed with explicit executive functions in mind, whereas we show that frontopolar cortex is involved in changes of implicitly learned configurations. 

### 3.4. Differentiation within Anterior Prefrontal Cortex?

**Figure 2:**
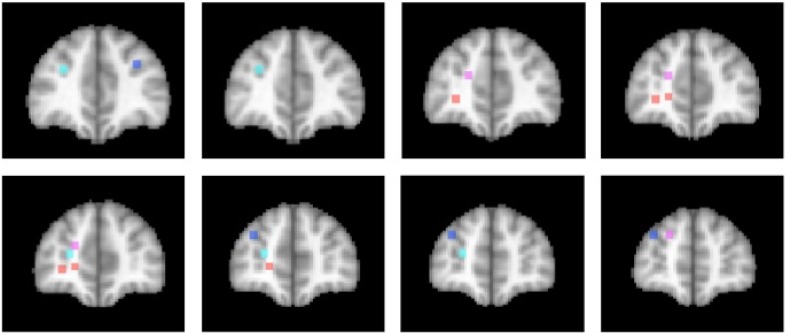
Activation locations in anterior prefrontal cortex. Dots indicate locations of activation in different studies of implicit attention control mechanisms. Color code: Contextual cueing (bluish colors): Blue: Target change early in learning [52]; Turquoise: Target change in repeated displays after extensive learning [46]. Cross-dimensional singleton search (reddish colors): Orange: Change-related activation [36,37]; Magenta: Dimensional uncertainty [36]. Left hemisphere is on the left. Slices represent 2 mm-spacings between Talairach [58]-coordinates *y* = 39 to *y* = 55.

The activations associated with change processes in the cross-dimensional visual search and contextual cueing paradigms were observed in an area that reaches from the banks of the frontomarginal sulcus (BA 10) to the banks of the superior frontal sulcus, at the border of Brodmann areas 10 and 9. Whereas the former was lateralized to the left hemisphere, the latter was left-dominant, but not exclusively observed in the left hemisphere. As of yet, it is an open question whether the more ventral frontomarginal sulcus and the more dorsal superior frontal sulcus regions subserve different functions. Figure 2 shows that target location changes in extensively repeated displays led to increased activations in both areas ([46]; turquoise dots), whereas the same changes in the early phase of learning elicited increased activation bilaterally along the banks of the superior frontal sulcus ([52]; blue dots). In the cross-dimensional search paradigm, dimension change-related activation was observed on the banks of the left frontomarginal gyrus ([36,37]; orange dots). Apart from the change-related activation, that was measured as event-related activation changes during change trials compared with same dimension trials, we also investigated the neural correlate of dimensional uncertainty. Dimensional uncertainty was measured as blockwise activation in blocks consisting of dimension change and same dimension trials, compared with blocks containing only same dimension trials. The activation increase that accompanied dimensional uncertainty ([36]; magenta dots) was more dorsal than the change-related activation, including the cortex along the banks of the superior frontal sulcus. Thus, the cortex surrounding the frontomarginal sulcus, particularly in the left hemisphere, may be involved particularly if contextual interference is‚ “detected” (though this need not become aware), whereas the cortex along the anterior part of the superior frontal sulcus may be involved in a more diffuse sense of uncertainty, that is independent of whether the actual trial contains a contextual interference/change or not. This uncertainty may also develop early in learning, when the association between a particular target stimulus and its context has not yet been strengthened enough. 

### 3.5. How Implicit Is Implicit Attention Control?

When we assume that anterior frontal cortex plays a role in implicit attention control, some caveats should be observed. Frontal activation in “implicit” tasks could be a mere by-product in that subjects, although unaware of the nature of cues such as repeated contexts may notice that they need longer in certain situations, that the task becomes more difficult, *etc*. In order to rule out such an explanation of frontopolar activation in visual dimension changes, we have carried out a study in patients with anterior prefrontal lesions [40]. We could show that patients with left lateral frontopolar lesions, in agreement with the activation data from neurologically normal subjects, who showed increased left lateral frontopolar activation following stimulus-driven dimension changes, showed a selective dimension change cost (*i.e*., longer search times for change trials). This finding speaks for an active contribution of left frontopolar cortex to visual dimension changes, it can hardly be explained if frontopolar activation was only an accessory phenomenon. 

Apart from that, explicit and implicit processes may be rather end points on a continuum than categorically different processes. In the contextual cueing paradigm, it has been criticized that often the statistical power of explicit memory tests used to infer the awareness of repeated display presentation was less than the power of the actual cueing experiment. When the power of the explicit memory test of display repetition was increased, it turned out that at least some of the displays had been explicitly learned [42,44]. Importantly, however, there was no correlation between the size of the search time reduction for repeated displays (the contextual cueing measure) and the degree of certainty with which a display could be judged as repeated. Thus, contextual cueing did not arise simply as a search advantage for a portion of displays that could be remembered explicitly. 

## 4. Conclusions

The data that we have discussed suggest a role of rostral prefrontal cortex in detecting violations of learned contingencies. This is different from the assumed role of, e.g., the TPJ area, which is supposed to support disengagement from the current focus of attention in response to any (typically salient) stimulus. The frontopolar cortex is in a well-suited position for this task, because it supports episodic memory retrieval [1,33] adding specifically an evaluative function [33,55]. In a sense, we also postulate an evaluative role of frontopolar cortex, in that the evaluation of the changed position of a target in a display that has been previously encountered is carried out without the individual becoming aware of it. 

A central difference between the tasks leading to rostral and more posterior lateral prefrontal cortex involvement appears to be that lateral prefrontal cortex supports executive processes in well-structured task sets, whereas rostral prefrontal cortex is involved in tasks without such a clear structure. For example, the metacontrast masked cues used to investigate unconscious task set selection or inhibition [13,15,16] did or did not reach consciousness, depending on the cue-mask interval, but they were associated with a clear instruction that did not change over the course of the experiment. In contrast, search displays in the contextual cueing tasks were consciously perceived, but the repetition of attention-guiding distractor configurations was not consciously noticed. Here, rostral prefrontal activation was observed when these learned contingencies changed and the repeated distractor configuration needed to be associated with a new target location for optimal search performance. This fits nicely with the literature on rostral prefrontal involvement in explicit processing of ill-structured situations [34,59]. 
